# Structural characterization of polysaccharides from *Cordyceps militaris* and their hypolipidemic effects in high fat diet fed mice†

**DOI:** 10.1039/c8ra09068h

**Published:** 2018-12-07

**Authors:** Zhen-feng Huang, Ming-long Zhang, Song Zhang, Ya-hui Wang, Xue-wen Jiang

**Affiliations:** School of Life Science, South China Normal University No. 55 West of Zhongshan Avenue Guangzhou Guangdong China zhangs@scnu.edu.cn

## Abstract

*Cordyceps militaris* is a crude dietary therapeutic mushroom with high nutritional and medicinal values. Mushroom-derived polysaccharides have been found to possess antihyperglycemic and antihyperlipidemic activities. This study aimed to partially clarify the structural characterization and comparatively evaluate hypolipidemic potentials of intracellular- (IPCM) and extracellular polysaccharides of *C. militaris* (EPCM) in high fat diet fed mice. Results indicated that IPCM-2 is α-pyran polysaccharide with an average molecular weight of 32.5 kDa, was mainly composed of mannose, glucose and galactose with mass percentages of 51.94%, 10.54%, and 37.25%, respectively. EPCM-2 is an α-pyran polysaccharide with an average molecular weight of 20 kDa that is mainly composed of mannose, glucose and galactose with mass percentages of 44.51%, 18.33%, and 35.38%, respectively. In *in vivo* study, EPCM-1 treatment (100 mg kg^−1^ d^−1^) showed potential effects on improving serum lipid profiles of hyperlipidemic mice, reflected by decreasing serum total cholesterol (TC), triglyceride (TG) and low density lipoprotein-cholesterol (LDL-C) levels by 20.05%, 45.45% and 52.63%, respectively, while IPCM-1 treatment (100 mg kg^−1^ d^−1^) remarkably decreased TC, TG and LDL-C levels by 20.74%, 47.93%, and 38.25%, respectively. In addition, EPCM-1 ameliorated hyperlipidemia possibly through upregulating the expression of serum lipoprotein lipase (LPL) and down-regulating the expression of hepatic 3-hydroxy-3-methylglutaryl-CoA reductase (HMGR), as determined by enzyme-linked immunosorbent assay (ELISA) method, while IPCM-1 remarkably upregulated the expression of serum LPL. This study confirms polysaccharides from *C. militaris* could be explored as functional foods or natural medicines for preventing hyperlipidemia.

## Abstract 2

Structural characterization and comparative evaluation of hypolipidemic activities of intracellular and extracellular polysaccharides from *Cordyceps militaris*.

## Graphical abstract

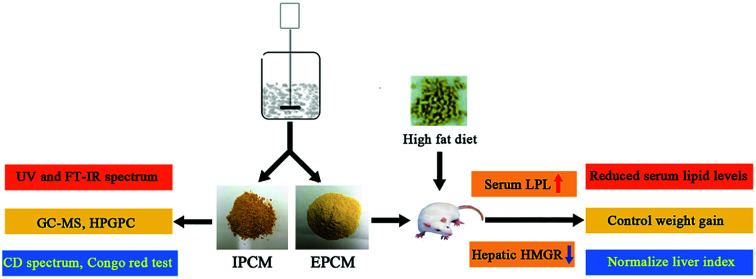

## Introduction

Hyperlipidemia is the most common risk factor for developing fatty liver and cardiovascular disease, which are major health problems and account for approximately one third of all deaths worldwide.^1^ Clinically, elevated serum total cholesterol (TC), triglyceride (TG), low density lipoprotein-cholesterol (LDL-C), very low density lipoprotein-cholesterol (VLDL-C) levels, and decreased high density lipoprotein-cholesterol (HDL-C) level are risk factors for hyperlipidemia.^2^ Up until now, current chemical drugs used to treat hyperlipidemia, statins (*e.g.*, lovastatin and simvastatin) and other hypolipidemic agents (*e.g.*, acipimox and fibrates), are known to have modest clinical efficacy. However, they have adverse effects such as rhabdomyolysis and hepatotoxicity, that limit their application.^3^ Due to the limited and unsatisfactory therapeutic effects of antihyperlipidemic agents, using functional foods and nutraceuticals has been an alternative treatment to manage hyperlipidemia.

Most, if not all, mushrooms contain polysaccharides with various biological activities in their fruiting body, cultured mycelia and culture broth. Due to their multiple and effective biological functions as well as nontoxic characteristics, mushroom-derived polysaccharides, a kind of natural biological macromolecule known as “biological response modifiers”, have received widespread clinical attention.^4,5^*Cordyceps militaris*, a valuable and rare edible-medicinal fungus belonging to the class Ascomycetes, has gained widespread popularity as a traditional medicine or nutraceutical due to its beneficial properties in healthy subjects, such as antitumor, immunomodulatory, antiinflammatory, antioxidative, hypouricemic, antinephritic, antihyperlipidemic, antibiotic and hepatoprotective effects.^6–11^ It contains multiple active components such as polysaccharides, cordycepin, adenosine, mannitol.^11^ As one of the major bioactive constituent of *C. militaris*, literature review shows that polysaccharides from *C. militaris*, exhibiting a potential for antioxidation, anti-inflammation and energy metabolism, are considered to possess beneficial effects on metabolic disorders such as hyperglycemia, hyperuricemic and hyperlipidemia, and therefore can function as novel potential hypolipidemic agents.^11–16^

Due to the advantages in more-compact culture space, shorter incubation time and higher production, currently, submerged fermentation has been used as a rapid and alternative method to accelerate the industrial level production of mushroom biomass and valuable metabolites.^17–19^ Recently, increasing evidences have indicated that the cordycepin and polysaccharides from fruiting bodies of cultured *C. militaris* show potential protection against hyperlipidemia,^11,20^ however, scarce literature about cultivated mycelia and fermented liquid has been published. Moreover, we found that extracellular polysaccharides released into culture medium during submerged fermentation of mushroom possessed a stronger biological properties than intracellular polysaccharides from mycelia in ours previous studies.^21^ To date, scarce investigation has been devoted to comparatively evaluate the hypolipidemic effects of polysaccharides from *C. militaris* mycelia and fermented liquid cultivated by submerged fermentation and underlying mechanisms. Furthermore, biological properties of polysaccharides were related with their molecular structure including monosaccharide composition, molecular weight, glycosidic bond, and conformation of the main chains.^17,19^ Gather up the threads, it is greatly significative and necessary to comparatively evaluate the effects of the intracellular- (IPCM) and the extracellular polysaccharides of *C. militaris* (EPCM) cultivated by submerged fermentation in preventing high-fat diet induced hyperlipidemia.

In this research, we focus on the preparation, structural characterization and hypolipidemic effects of polysaccharides from *C. militaris*. Firstly, two crude polysaccharides, IPCM-1 and EPCM-1, were isolated from the mycelia and fermented liquid of *C. militaris*, respectively. Afterward, two purified polysaccharides were obtained by column chromatography purification and characterized by Fourier transform-infrared spectroscopy (FT-IR), high performance liquid gel permeation chromatography (HPGPC), gas chromatography-mass (GC-MS) and circular dichroism (CD). In addition, the hypolipidemic activities of polysaccharides were evaluated using high-fat diet fed mice as model for exploration of clinical antihyperlipidemic mechanisms in pharmaceutical industry.

## Materials and methods

### Materials

Diethylaminoethyl (DEAE) −52 cellulose and Sephadex G-100 were obtained from Whatman. Co. (British). Dialysis tubes were obtained from Guangzhou Dongju Experimental Apparatus Co. Ltd. (Guangzhou, China). Trypsin 1 : 250 from porcine pancreas was purchased from Biosharp Biological Technology Co., Ltd (Hefei, China). Bovine serum albumin (BSA) and vitamin B_1_ were provided by Hangzhou Sijiqing Biological Engineering Materials Co. Ltd. (Hangzhou, China). All the other chemicals were of analytical grade.

### Fermentation process

*C. militaris* used in the present study was donated by College of Life Science, South China Normal University (Guangzhou, Guangdong, China). The strain was maintained on slant medium (potato 200 g, glucose 20 g, peptone 1.0 g, (NH_4_)_2_SO_4_ 1.0 g, KH_2_PO_4_ 1.0 g, MgSO_4_·7H_2_O 1.0 g and agar 20 g per liter of distilled water, pH 6.5), and incubated at 24 °C for 8 days. The *C. militaris* mycelia were transferred to 200 mL of sterile seed culture medium on a rotary shaker incubator at 26 °C and 160 rpm to cultivate for 6 days. The seed medium and fermentation medium contained glucose (50 g L^−1^), KH_2_PO_4_ (2.0 g L^−1^), NH_4_NO_3_ (2.0 g L^−1^), MgSO_4_·7H_2_O (1.0 g L^−1^), and vitamin B_1_ (0.05 g L^−1^). Seed culture obtained was cultivated at 27 °C in a 50 L fermentor (Baiou Equipment Co. Ltd, Guangzhou, China). The entire fermentor with 30 L fermentation medium was autoclaved at 121 °C for 30 min. Fermentations were performed according the following conditions: initial pH, 6.5; inoculum size, 7% (v/v); temperature, 27 °C; aeration rate, 1 vvm; rotation speed, 150 rpm; tank pressure, 0.05–0.07 MPa; working time, 96 h. The bioreactor culture obtained was transferred to a 500 L fermenter (Baiou Equipment Co. Ltd, Guangzhou, China) containing 300 L fermentation medium under the same conditions described above for another 96 h.

### Extraction and characterization of IPCM-1 and EPCM-1

Bioreactor culture obtained as described above was carried in ultra-speed centrifuge (GF-105 (B), Guangzhou GuangZhong Enterprise Group Corp, Guangzhou, China) to centrifuge at 15 000 rpm for 20 min to separately collect *C. militaris* mycelia and fermentation supernatant.

Fermentation supernatant was ultra-filtrated (polysulfone ultrafiltration membrane with a molecular cut off weight of 10 kDa, HeFei ShiJie Membrane Engineering Co. Ltd, Hefei, China) to remove low-molecular-weight compounds (operating conditions: pressure, 10 bar; flux, 3–5 L min^−1^; temperature, 22 °C), followed by rotary evaporation under reduced pressure at 60 °C for further concentration. The broth culture filtrate was used to extract the EPCM-1. Mycelia were gathered, dried, crashed, sieved and defatted with 1 : 2 (w/v) of petroleum ether. After drying at 50 °C, mycelia were used to extract the IPCM-1.

The two polysaccharides were prepared according a previously reported method with some modification.^22^ The fermented liquid obtained were used to remove protein thoroughly by a combination of trypsin enzymolysis and Sevag method.^23^ Then the supernatant liquids were precipitated with 4 times volume of 95% (v/v) ethanol, depigmented with 30% (v/v) H_2_O_2_, and dialyzed against distilled water (6000–8000 Da molecular weight cut-off) for 3 days to obtain the EPCM-1. The mycelia were extracted three times with hot distilled water (1 : 25, w/v) at 75 °C for 2.5 h followed by the purification processes described above for IPCM-1. A yield of 8.33% (lyophilized weight) water-soluble crude polysaccharides IPCM-1 were isolated from *C. militaris* mycelia, while crude polysaccharides EPCM-1 were prepared in a yield of 52.4 mg L^−1^ from fermented liquid.

### Purification of IPCM-1 and EPCM-1

Approximately 100 mg of crude polysaccharides dissolved in 10 mL of Tris–HCl solution (pH 8.0) were loaded on a DEAE-52 cellulose column (3.0 cm × 80 cm), which were pre-equilibrated with Tris–HCl solution, and eluted in 0.4 M NaCl–Tris solution (pH 7.2) at a flow rate of 1 mL min^−1^ (6 mL per tube). The products obtained were further purified by size-exclusion chromatography on a Sephadex G-100 column (3.0 cm × 80 cm) and eluted with 1 M NaCl–Tris solution (pH 8.0) at a flow rate of 1 mL min^−1^ (6 mL per tube). Each elute was monitored for carbohydrate determination *via* using the phenol-sulfuric acid method with d-glucose as a standard.^24^ The same carbohydrate-positive fractions were pooled together, dialyzed, and lyophilized. The lyophilized samples of two purified polysaccharides, termed IPCM-2 and EPCM-2, were white powders and they showed positive responses to Molisch test, and negative reactions to iodine-potassium iodide, Fehling's reagent and ferric trichloride, ninhydrin and Bradford test, which indicates the absence of starch-type polysaccharides, reducing sugars and phenolics, amino acids and proteins in IPCM-2 and EPCM-2.^23,25^ The percentage of total carbohydrate content was 91.63% in IPCM-2 and 91.68% in EPCM-2.

### UV and FT-IR spectrometric analysis

The polysaccharide solution (1 mg mL^−1^) was prepared and applied to ultraviolet spectral analysis. The UV scanning spectrum was measured in the wavelength range of 190–600 nm by spectrophotometer (UV-2400, Shimadzu, Japan).

The absorption spectrum of the IPCM-2 and EPCM-2 were obtained using FT-IR spectroscopy. 10 mg of the sample was thoroughly ground with KBr powder (100 mg). The dried mixture was then pressed into 1 mm thick pellets for FT-IR spectral analysis. The FT-IR spectra were recorded in a range of 4000–450 cm^−1^ by an transmission infrared spectrometer (Sigma, USA).

### Molecular weight distribution analysis

The molecular weight and homogeneity of IPCM-2 and EPCM-2 were measured by HPGPC, which was operated with a HPLC system (1100, Agilent Technologies, USA) equipped with a refractive index detector (RID, detecting temperate 35 °C) and a TSK-GEL G3000SW XL column (300 mm × 718 mm).^19^ NaH_2_PO_4_–Na_2_HPO_4_ aqueous solution (0.05 M, pH 6.7) was used as mobile phase at a flow rate of 0.8 mL min^−1^. Standard dextrans with different molecular weights (*M* 5.8, 12.2, 23.7, 48, 100, 186, 380, 853 kDa) were used for comparison to calculate the standard curve. The relationship between log *M* and retention time *T* was calculated as the following equation:

1 log *M* = − 0.2524*T* + 7.5766 *R*^2^ = 0.982

### Monosaccharide composition analysis

GC-MS spectrometry was applied for the identification and quantification of monosaccharides of IPCM-2 and EPCM-2.^26^ Briefly, 2 M sulfuric acid was used to hydrolyze polysaccharide at 100 °C for 6 h. Barium hydroxide was used to adjust pH of hydrolyzed polysaccharide to neutrality and then the mixture was evaporated at 45 °C. The hydrolysate was dissolved in 5 mL of pyridine with 70 mg of hydroxylamine hydrochloride in a tube and reacted at 90 °C for 1 h. After cooling, 5 mL of acetic anhydride was added and the mixture was incubated at 90 °C for 1 h, then analyzed by GC-MS on a Agilent 6890GC/5973MS chromatograph equipped with a fused silica capillary column (15 mm × 0.2 mm). The standard chromatograms of ribose, rhamnose, arabinose, xylose, mannose, glucose and galactose were used for composition identification of IPCM-2 and EPCM-2 (chemical structures of monosaccharides see Fig. S1†), and the relative mass percentages were calculated by the area normalization method.

### CD spectrum analysis

CD spectrum was employed to analyze the conformation IPCM-2 and EPCM-2.^27^ The polysaccharide samples dissolved in deionized water (0.05 mg mL^−1^) was kept at 25 °C for 2 h. The mixtures were analyzed by CD spectrometer (J-500, Jasco Corporation, Japan). Data was collected in a range of 190–350 nm at 1 nm interval.

### Congo red analysis

The conformational structures of the polysaccharides were determined by Congo red analysis referenced reported method.^28^ Firstly, polysaccharide solution (2.5 mg mL^−1^) was mixed with the same volume of Congo red aqueous solution (80 μM). Then, NaOH solution (1 M) were added to bring a final NaOH concentrations to 0, 0.10, 0.20, 0.30, 0.40 and 0.50 M. After incubating at 25 °C for 10 min, these mixtures were employed to ultraviolet spectral analysis in the wavelength range of 400–800 nm find the *λ*_max_ (maximum absorption wavelength). The process above was carried out using pure water instead of polysaccharide solution as a control.

### Animals, diets, and experimental procedures

**Fig. 1:**
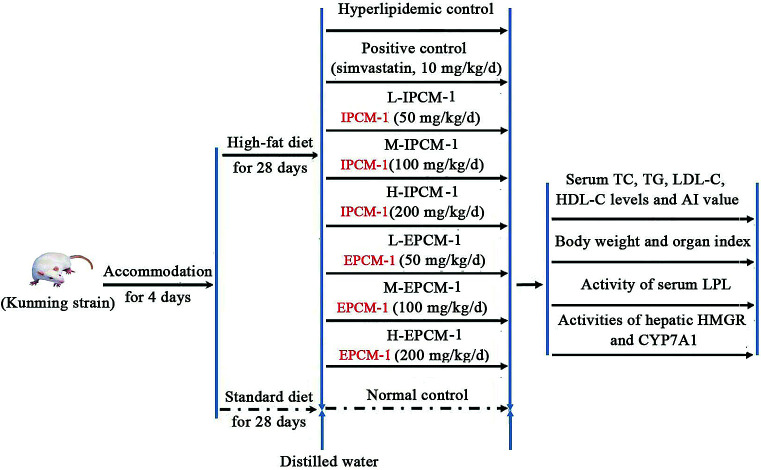
Animal experimental procedure.

Allowing for the low efficiency and yield of column chromatographic purification, and the large amount of IPCM-2 and EPCM-2 in IPCM-1 and EPCM-1, respectively, a large dosage of IPCM-1 and EPCM-1 were used to investigate hypolipidemic activities of IPCM-2 and EPCM-2 in mice fed a high-fat diet.^29^ Ninety adult male Kunming (KM) mice, weighting 20 ± 2 g, were obtained from the Experimental Animal Center of Sun Yat-sen University (Guangzhou, Guangdong, China) [Certificate No. SCXK (Yue) 2011–0029]. During the experiment, the mice were housed in individual stainless-steel cages and maintained at 20 ± 2 °C under a light/dark cycle of 12 h and relative humidity 55 ± 5%. All mice were acclimated to laboratory conditions for 4 days before the onset of the experiment and they had free access to rodent laboratory food and drinking water. Simvastatin tablet from Hangzhou Merck & Co., Inc. (Approval number: J20090001) was used as a positive control drug.^17,19^ The mice as mentioned above were randomly divided into 9 groups, with 10 mice in each group received the following treatment schedule (Fig. 1):

Normal control (NC), mice fed a standard diet with daily oral gavage 10 mL kg^−1^ body weight (BW) distilled water.

Hyperlipidemic control (HC), mice fed a high-fat diet with oral gavage 10 mL kg^−1^ BW distilled water daily.

Positive control (PC), fed with high-fat diet and received in addition 10 mg kg^−1^ BW of simvastatin daily.

L-IPCM-1, M-IPCM-1 and H-IPCM-1: mice fed a high-fat diet and supplemented with IPCM-1 through daily gavage at doses of 50, 100, and 200 mg kg^−1^ BW, respectively.

L-EPCM-1, M-EPCM-1 and H-EPCM-1: mice fed a high-fat diet and supplemented with EPCM-1 through daily gavage at doses of 50, 100, and 200 mg kg^−1^ BW, respectively.

The feed for the experimental animals was supplied by Guangdong Medical Laboratory Animal Center (Guangzhou, Guangdong, China) [Certificate No. SCXK (Yue) 2008–0002]. The high-fat diet consisted of 1.0% cholesterol (w/w), 10% lard, 0.3% bile salt, and 88.7% standard diet. The animal experiment was lasted for 28 consecutive days. Body weight was measured weekly and food intake adjusted until the end of the experiment. At the end of experiment, all mice were fasted overnight and sacrificed by clavicle dislocation after anesthetizing with sodium thiopental. Blood samples were collected in heparinized tubes and immediately separated for acquisition of serum by centrifugation at 4 °C and 3000 rpm for 15 min, and then stored at −80 °C until biochemical analysis. After blood collection, the heart, liver, kidney and spleen were excised immediately, wiped by filter paper and weighed after being rinsed with ice-cold phosphate buffered saline (PBS). Organ indexes were defined as the weight of organs divided by the weight of mice. Liver tissue was homogenized with ice-cold PBS and ten percent (w/v) of liver homogenate was centrifuged at 4 °C and 4000 rpm for 10 min. The liver supernatant obtained after centrifugation twice was stored at −20 °C until assay.

All experimental procedures were performed in accordance with the Guidelines for Animal Care established by the National Institute of Health, and approved by the Ethics Committee for animal research at South China Normal University.

### Determination of serum lipid profiles

Serum lipids level indices of TC, TG, LDL-C, and HDL-C were measured using commercial assay kits purchased from Nanjing JianCheng Institute of Bioengineering (Nanjing, China). TC and TG levels were quantified with colorimetric enzymatic method by the commercial kit CHOD-PAP-enzymatic-colorimetric and GPO-PAP-enzymatic-colorimetric, respectively.^30^ HDL-C and LDL-C levels were measured using direct enzymatic commercial assay kits.^31^ The results were expressed in mmol L^−1^ of serum. The atherogenic index (AI) was calculated as the following formula:^32^2
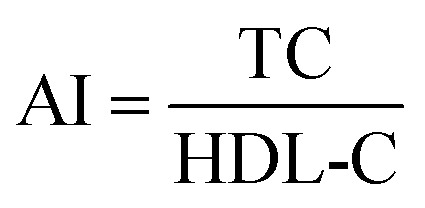


### Determination of serum LPL activity

LPL can catalyze the decomposition of core TG in chylomicrons and VLDL-C into free fatty acids.^33^ The activity of serum LPL was measured using commercial assay kits purchased from the Nanjing Jiancheng Institute of Bioengineering (Nanjing, Jiangsu, China) according to the manufacturer's instructions, which was quantified based on the free fatty acid production. Briefly, the sample was reacted with substrate solution of assay kits at 37 °C for 20 min, then the content of free fatty acids of samples was detected with copper reagent method by iMARK Microplate Reader (Bio-Rad Laboratories, Hercules, CA, USA), which have a maximum absorbance at 550 nm in UV-vis spectrophotometer. The controls were treated with the same quantity of double distilled water. The activity of serum LPL (*A*_LPL_) was calculated using the following equation:3
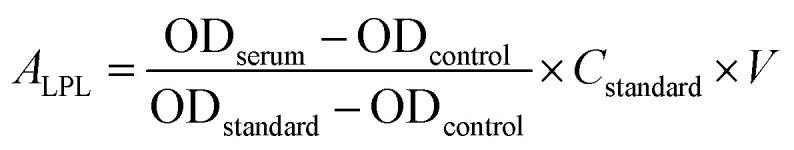
where OD_serum_, OD_control_ and OD_standard_ are the optical density (OD) value at 550 nm of the serum sample, the control and LPL standard, respectively, *C*_standard_ is the concentration of LPL standard (0.5 μmol mL^−1^) and *V* is the volume of the sample (mL). *A*_LPL_ was recorded in U mL^−1^ of serum. One unit of LPL activity was defined as 1 μmol of free fatty acids released in 1 mL of serum with a 1 h reaction time.

### Determination of hepatic HMGR and CYP7A1 activities by sandwich enzyme-linked immunosorbent assay (ELISA)

Hepatic HMGR and CYP7A1 activities were measured using standard sandwich ELISA kits (R&D, USA) according to the manufacturer's instructions. Briefly, assay plates were coated with mouse anti-HMGR or anti-CYP7A1 antibody and blocked with 5% skim milk at 25 °C for 2 h. Then the plates were reacted with 50 μL liver supernatant samples diluted with diluent solution (1 : 4 v/v) at 37 °C. After reactions for 30 min, the plates were washed five times using the wash buffer. The captured HMGR or CYP7A1 was detected using biotin-antibody, together with horseradish peroxidase (HRP)-conjugated avidin. After washing five times with the wash buffer, color reactions were reacted with 3,3′,5,5′-tetramethylbenzidine (TMB) substrate and measured at 450 nm. Two enzyme standards with different concentration were used to calibrate the standard curve. HMGR and CYP7A1 activities of samples were determined according to the standard curves and the results were recorded in U L^−1^ of liver supernatant.

### Statistical analysis

The data were expressed as means ± standard deviation (SD) and determinations were obtained from ten animals per group. The one-way analysis of variance (ANOVA) followed by Student's *t*-test was used to detect significant differences between any two groups. *P*-value < 0.05 were regarded as statistically significance.

## Results and discussion

### Spectrometric analysis

**Fig. 2:**
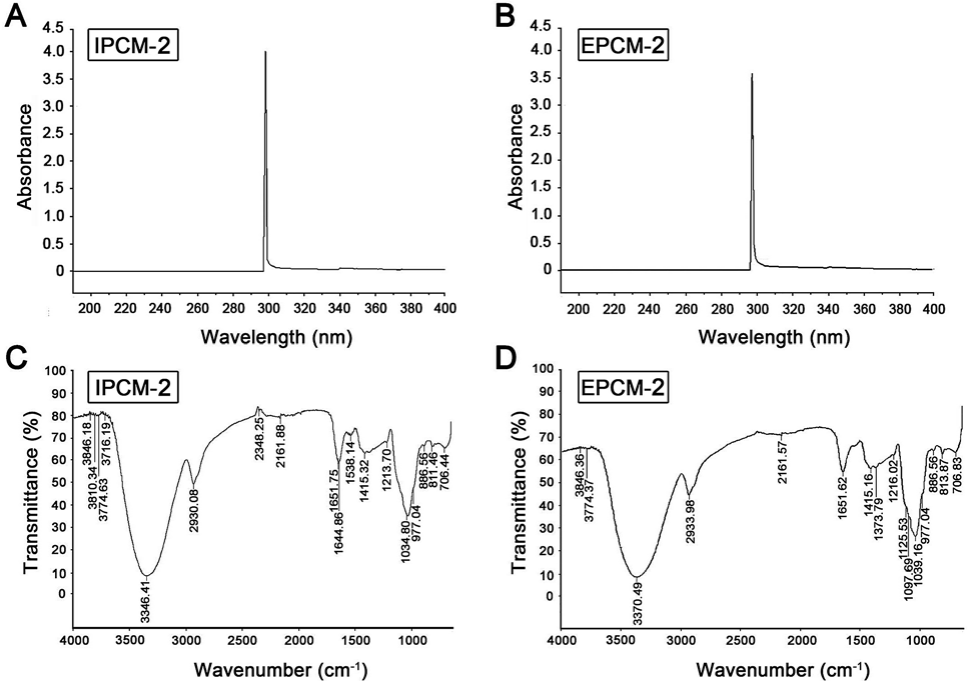
Spectrometric analysis. UV spectrum of IPCM-2 (A) and EPCM-2 (B); FT-IR spectrum of IPCM-2 (C) and EPCM-2 (D).

The UV spectrum of IPCM-2 and EPCM-2 were presented in Fig. 2A and B. No absorption was occurred at 260 and 280 nm in the ultraviolet spectrum of IPCM-2 and EPCM-2, indicating absence of nucleic acids and protein contained.^19^ The results were exactly consistent with the determination in protein content by Bradford assay and further showed that DEAE-52 cellulose column selected were effective.

The FT-IR spectroscopy of IPCM-2 and EPCM-2 were showed in Fig. 2C and D. We can find that IPCM-2 and EPCM-2 showed similar absorption bands in the 450–4000 cm^−1^ region which are admitted as the characteristic peaks of polysaccharides.^17,26^ Specifically, the broad stretching peaks at 3346 (IPCM-2) and 3370 (EPCM-2) cm^−1^ were ascribed to the hydroxyl groups with stretching vibration. The absorption bands at about 2930 cm^−1^ (IPCM-2) and 2933 cm^−1^ (EPCM-2) were attributed to C–H stretching vibrations of the polysaccharides. The strong band at 1651 cm^−1^ was derived from C=O vibration, and the weak bond at about 1415 cm^−1^ was originated from C–H bending vibration. The two bonds at 1034 cm^−1^ (IPCM-2) and 1039 cm^−1^ (EPCM-2) were the characteristic of C–O–C, suggesting the presence of pyranose sugars. Moreover, characterization of IPCM-2 and IPCM-2 showed the typical absorption of D-pyran ring grape asymmetric stretching vibration at round 977 cm^−1^ and symmetric stretching vibration at round 706 cm^−1^. The absorption bands centered at 811 (IPCM-2) and 813 (EPCM-2) cm^−1^ were due to the α-type glycosidic linkages. Thus, IPCM-2 and EPCM-2 are both α-pyran polysaccharides.

### Molecular weight and monosaccharide composition

**Fig. 3:**
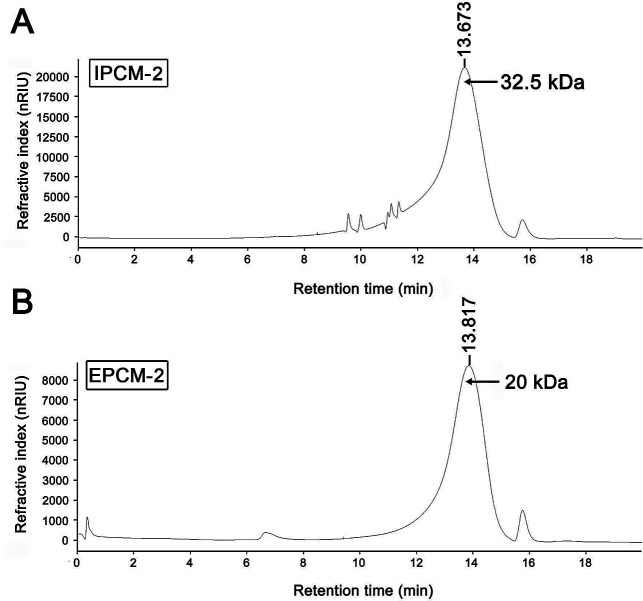
High performance liquid gel permeation chromatography chromatogram of IPCM-2 (A) and EPCM-2 (B).

As shown in Fig. 3, a single symmetrical peak was found in the HPGPC profiles of IPCM-2 and EPCM-2, indicating that they are both homogeneous polysaccharides. The weight-average molecular weight (*M*_w_) and number-average molecular weight (*M*_n_) of IPCM-2 was 32.5 and 16.1 kDa, respectively. The *M*_w_ and *M*_n_ of EPCM-2 was 20 and 13.4 kDa, respectively. The *M*_w_/*M*_n_ values of IPCM-2 and EPCM-2 were 2.02 and 1.49, respectively, suggesting that IPCM-2 and EPCM-2 are polysaccharide polymers.

**Fig. 4:**
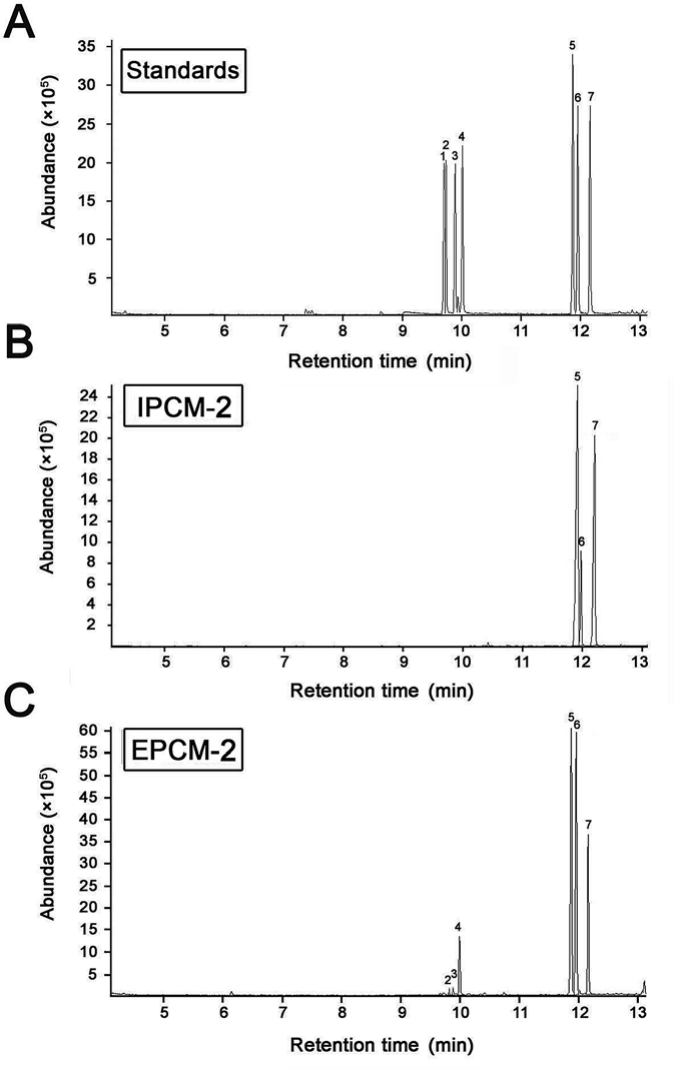
Gas chromatography-mass chromatograms of monosaccharide standard samples (A), IPCM-2 (B) and EPCM-2 (C). Peaks: (1) ribose, (2) rhamnose, (3) arabinose, (4) xylose, (5) mannose, (6) glucose and (7) galactose.

The monosaccharide compositions of IPCM-2 and EPCM-2 were analyzed by means of comparing the retention time with monosaccharide guide samples (Fig. 4). The IPCM-2 was composed of seven different monosaccharides including ribose, rhamnose, arabinose, xylose, mannose, glucose and galactose with a mass percentage of 0.012%, 0.066%, 0.11%, 0.08%, 51.94%, 10.54% and 37.25%, respectively, while the EPCM-2 was composed of ribose, rhamnose, arabinose, xylose, mannose, glucose and galactose in a percentage composition of 0.14%, 0.35%, 0.57%, 0.72%, 44.51%, 18.33% and 35.38%, respectively. Obviously, the main difference between the two polysaccharides is the amount of glucose, whose content in EPCM-2 is markedly higher than that in IPCM-2. The monosaccharide compositions analysis revealed that the major components of IPCM-2 and EPCM-2 were mannose, glucose and galactose, and minor contents of ribose, rhamnose, arabinose and xylose were also noted, which was consistent with previous studies.^4,5^

### Spatial conformation studies

**Fig. 5:**
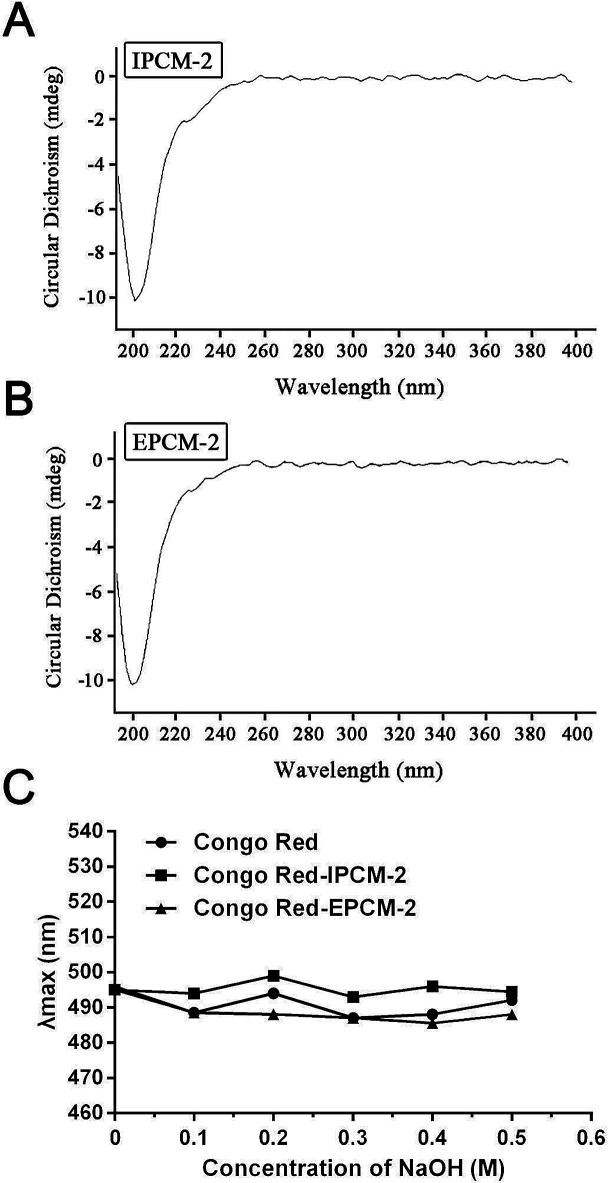
Conformation studies of IPCM-2 and EPCM-2. (A): Circular dichroism spectra of IPCM-2; (B): circular dichroism spectra of EPCM-2; (C): result of the maximum absorption wavelength (*λ*_max_) of Congo red test.

From the results of the CD spectra (Fig. 5A and B), CD signals of IPCM-2 and EPCM-2 appeared at 200 nm, showed negative cotton effect, indicating that IPCM-2 and EPCM-2 were asymmetric molecules. Congo red can form complexes with a helical conformation of polysaccharides when Congo red is mixed under alkaline conditions, and *λ*_max_ of the complex will show bathochromic shift in the wavelength range of 400–600 nm as compared with pure Congo red.^34^ However, the *λ*_max_ of IPCM-2- and EPCM-2-Congo red complexes had no bathochromic shift compared with control group, indicating that IPCM-2 and EPCM-2 did not exhibit a triple-helical conformation at alkali concentrations of 0 to 0.5 M and showed random coil conformations (Fig. 5C). These results were in accordance with Lee's reports.^4,5^

### Effects of IPCM-1 and EPCM-1 on serum lipid profiles

**Fig. 6:**
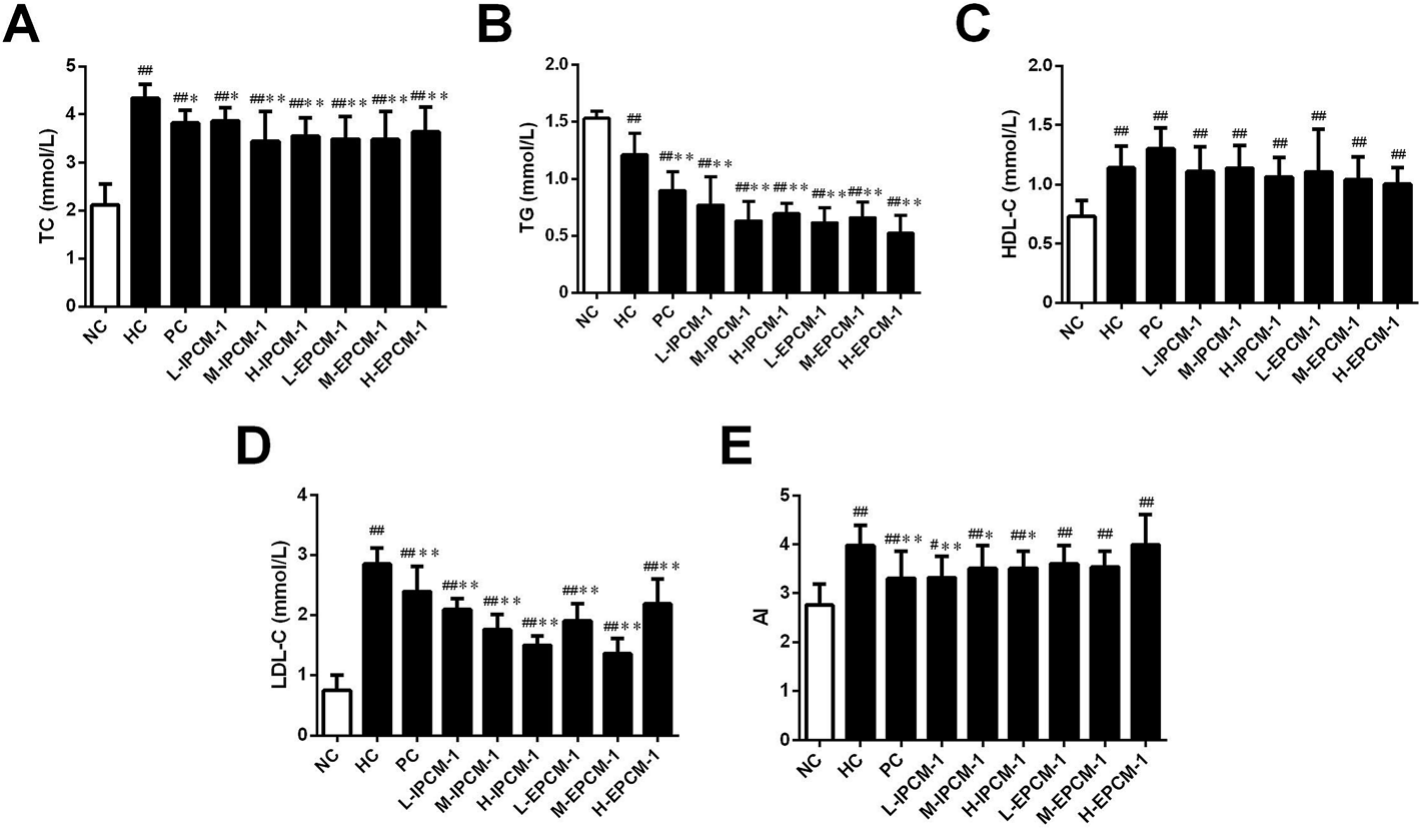
Effects of IPCM-1 and EPCM-1 on serum lipid profiles in hyperlipidemic KM mice. (A) Total cholesterol (TC), (B) triglyceride (TG), (C) high density lipoprotein-cholesterol (HDL-C), (D) low density lipoprotein-cholesterol (LDL-C), (E) atherosclerosis index (AI) value. Groups: normal control (NC), treated with standard diet; hyperlipidemic control (HC), treated with high-fat diet; positive control (PC), treated with high-fat diet and simvastatin (10 mg kg^−1^ d^−1^); L-IPCM-1, M-IPCM-1 and H-IPCM-1, treated with high-fat diet and IPCM-1 at 50, 100 and 200 mg kg^−1^ d^−1^, respectively; L-EPCM-1, M-EPCM-1 and H-EPCM-1, treated with high-fat diet and EPCM-1 at 50, 100 and 200 mg kg^−1^ d^−1^ at 50, 100 and 200 mg kg^−1^ d^−1^, respectively. Data are presented as means ± SD, *n* = 10. #*p* < 0.05 and ##*p* < 0.01, values *versus* NC group values, *p < 0.05 and **p < 0.01, values *versus* HC group values.

High TC and TG are risk factors that can cause hyperlipidemia and several literatures have reported that alleviating the elevated serum TC and TG levels is quite beneficial for the amelioration of hyperlipidemia.^17^ In addition, elevated serum TG and LDL-C levels are proposed to be related with increased risk of coronary heart disease.^35^ Effects of IPCM-1 and EPCM-1 on serum lipid profiles of hyperlipidemic mice were examined and results are shown in Fig. 6. It is clear that compared with NC mice, the serum levels of TC, HDL-C and LDL-C in HC mice were significantly higher, whereas the serum TG level was remarkably lower presumably because dietary administration of high-fat diet cause non-alcoholic fatty liver disease (NAFLD), which involves increased TG uptake and decreased lipid export in the liver.^36^ It indicated that high-fat diet feeding resulted in obvious lipid metabolism disorder in the KM mice and made them hyperlipidemia mice. *In vivo* study, positive simvastatin, a high efficiency antihyperlipidemic drug, was manifested potential effects against the increase of serum TC (11.98% decrease, *p* < 0.05), TG (25.62% decrease, *p* < 0.01) and LDL-C (16.14% decrease, *p* < 0.01) levels induced by high-fat diet, whereas no obvious effect in HDL-C level. Interestingly, the serum levels of TC, TG and LDL-C in the IPCM-1 and EPCM-1 treated group were remarkably lower than in the PC group. As presented in Fig. 6, in the dosage groups treated with IPCM-1 at 50, 100 and 200 mg kg^−1^ d^−1^, the serum TC level reached 11.06% (*p* < 0.05), 20.74% (*p* < 0.01), and 18.43% (*p* < 0.01) lower than that in the HC group, respectively (Fig. 6A). EPCM-1 treatment at three dosages caused significant (*p* < 0.01) decrease in serum TC level by 19.82%, 20.05% and 16.36%, respectively. Meanwhile, for concentrations of 50–200 mg kg^−1^ d^−1^, there were distinct (*p* < 0.01) reduction in TG level in mice treated with IPCM-1 by 37.19%, 47.93%, and 42.15%, respectively, and with EPCM-1 by 49.59%, 45.45%, and 57.02%, respectively, compared with HC group (Fig. 6B). Regarding LDL-C level, dietary supplement of IPCM-1 at the dosage of 50, 100, and 200 mg kg^−1^ d^−1^ caused a significant (*p* < 0.01) reduction in dose-dependent manners by 26.32%, 38.25%, and 47.37%, respectively, and administration of EPCM-1 at three dosages led a significant (*p* < 0.01) reduction by 33.33%, 52.63%, and 21.75% decrease, respectively, compared with that in HC group (Fig. 6D), thereby alleviating atherosclerotic lesions. However, no significant change in HDL-C level was observed both in IPCM-1- and EPCM-1- treated groups, indicating TC can not be transferred from peripheral tissues to the liver *via* the reverse cholesterol transport pathway (Fig. 6C). More importantly, we found that at the optimal dosage, IPCM-1 (100 mg kg^−1^ d^−1^) and EPCM-1 (100 mg kg^−1^ d^−1^) exerted the superior effects on reduction in serum lipid than simvastatin. The hypolipidemic activities of IPCM-1 and EPCM-1 were approximate two-fold as effective as simvastatin. Recent studies revealed that polysaccharides with relatively low molecular weight have higher biological activities.^37–39^ Moreover, numerous studies have demonstrated that content of glucose plays a key role in the improvement of hyperlipidemia.^17–19^ Owing to the large amount of IPCM-2 and EPCM-2 in IPCM-1 and EPCM-1, the bioactivity of IPCM-1 and EPCM-1 were mainly caused by IPCM-2 and EPCM-2. Combining the results of characterization of IPCM-2 and EPCM-2, we speculated that the relatively low molecular weight and the more content of glucose may explain the reason why EPCM-1 exhibited greater hypolipidemic activities than IPCM-1. Besides, AI value is an indicators of atherosclerosis and cardiovascular disease risk.^32^ Compared with hyperlipidemic control, IPCM-1 application showed a significant (*p* < 0.05) reduction in AI values, while dietary supplement of EPCM-1 resulted in weak reduction in AI values with no statistical significant difference (Fig. 6E).

These results are in agreement with the work of Wang and Kim, who reported that dietary supplementation of polysaccharides isolated from *C. militaris* fruiting bodies or residue could improve serum lipid profiles and serve as a protection or prevention of hyperlipidemia.^11,12^ Similarly, Dong and Yang also reported that polysaccharide-enriched fraction of *C. militaris* mycelia or culture supernatant in submerged fermentation could ameliorate the increase of serum lipid levels in hyperlipidemic rats.^40,41^ Consistent with previous works, IPCM-1 and EPCM-1 possess superior hypolipidemic properties than positive simvastatin.

### Effects of IPCM-1 and EPCM-1 on body weight and organ weight

**Fig. 7:**
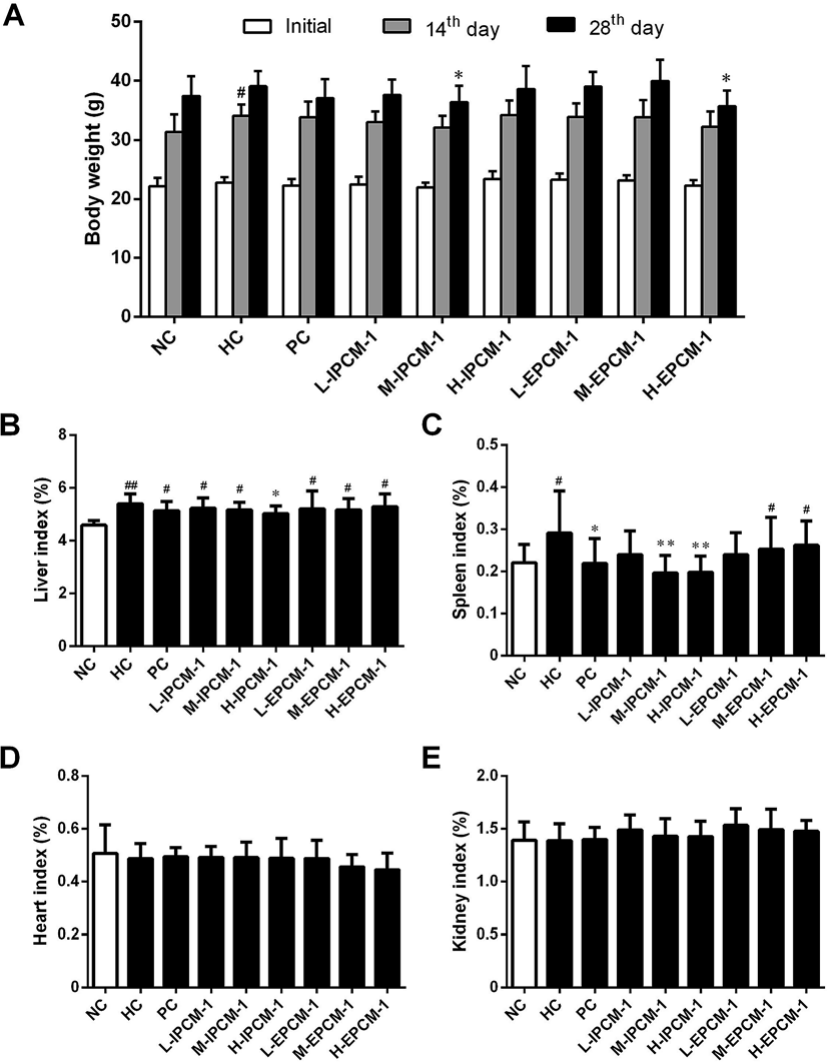
Effects of IPCM-1 and EPCM-1 on body weight and organ index in hyperlipidemic KM mice. (A) Body weight, (B) liver index, (C) spleen index, (D) heart index, (E) kidney index. Groups: normal control (NC), treated with standard diet; hyperlipidemic control (HC), treated with high-fat diet; positive control (PC), treated with high-fat diet and simvastatin (10 mg kg^−1^ d^−1^); L-IPCM-1, M-IPCM-1 and H-IPCM-1, treated with high-fat diet and IPCM-1 at 50, 100 and 200 mg kg^−1^ d^−1^, respectively; L-EPCM-1, M-EPCM-1 and H-EPCM-1, treated with high-fat diet and EPCM-1 at 50, 100 and 200 mg kg^−1^ d^−1^ at 50, 100 and 200 mg kg^−1^ d^−1^, respectively. Data are presented as means ± SD, *n* = 10. #*p* < 0.05 and ##*p* < 0.01, values *versus* NC group values, *p < 0.05 and **p < 0.01, values *versus* HC group values.

Body weight is considered as a putative indicator of health. Excess weight gain is often associated with increased fat mass, which was caused by hypertrophic and hyperplastic growth of adipocytes.^42^ As shown in Fig. 7A, no significant difference was detected in initial body weight of mice. However, the body weight in the HC mice was significantly (*p* < 0.05) higher than that in the NC mice after feeding high-fat diet for 14 days, but with no significant difference for 28 days presumably because of the unpleasant taste of high-fat diet. As compared with that in HC group, the mice treated with IPCM-1 (100 mg kg^−1^ d^−1^) and EPCM-1 (200 mg kg^−1^ d^−1^) for 28 days were less weight gain. These results clearly revealed that IPCM-1 and EPCM-1 were capable in control of the weight gain induced by high-fat diet, indicating that they could be applied as a future drug for weight loss. Liver is important organ for lipid metabolism. In the present study, liver indexes of HC mice were significantly higher than NC mice (*p* < 0.01) possibly because high-fat diet caused lipid accumulation in the liver.^12,36^ H-IPCM treatment trigger observable (*p* < 0.05) reduction in liver index as hepatoprotective action (Fig. 7B). Besides, IPCM-1 was also manifested distinct effects against the increase of spleen index induced by high-fat diet, which indicates that IPCM-1 exerted significant promotion with both the liver and spleen indexes (Fig. 7C). No significant difference in the organ indexes of heart and kidney was observed as a consequence of the dietary supplement of IPCM-1 and EPCM-1 as compared with HC mice (Fig. 7D and E).

### Effects of IPCM-1 and EPCM-1 on lipid regulating enzymes activities

**Fig. 8:**
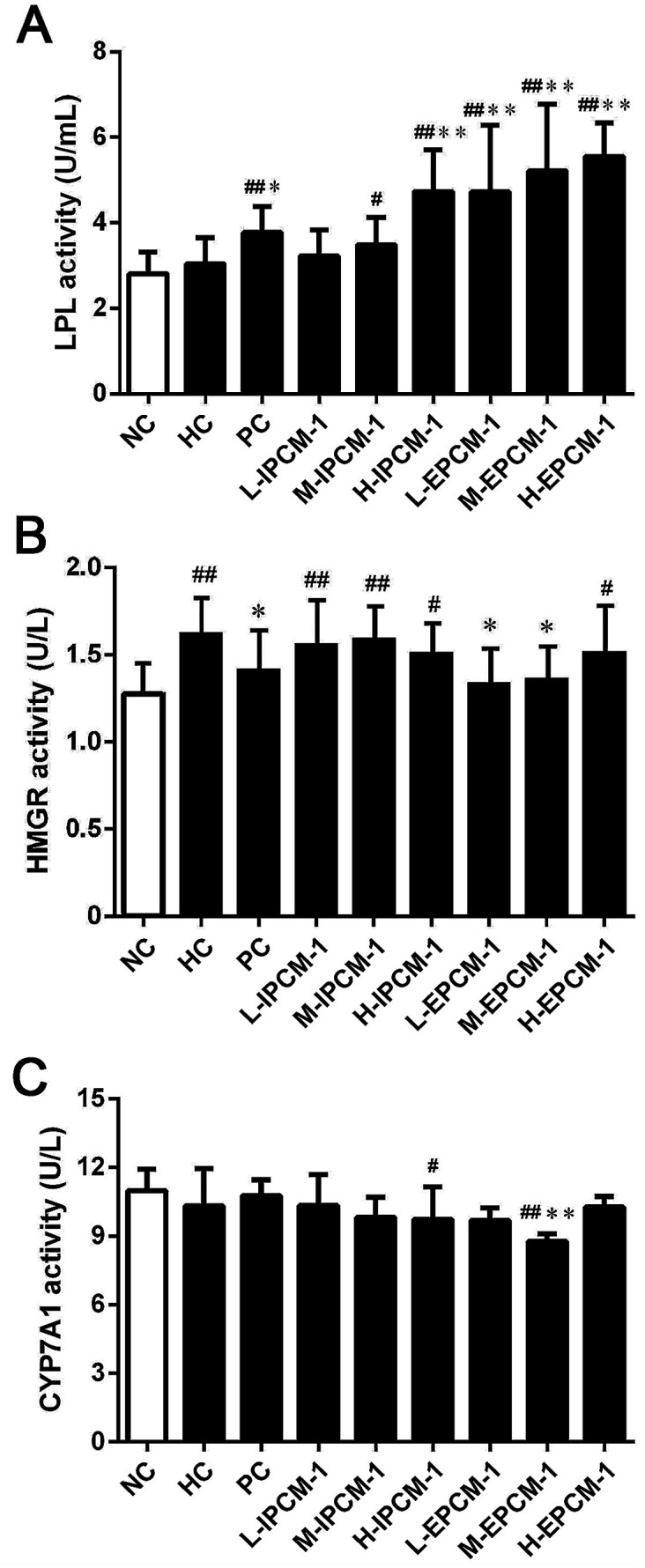
Effects of IPCM-1 and EPCM-1 on serum lipoprotein lipase (LPL) activity (A), hepatic 3-hydroxy-3-methylglutaryl-CoA reductase (HMGR) activity (B) and cholesterol 7-alpha-hydroxylase (CYP7A1) activity (C) of KM mice fed a high-fat diet. Groups: normal control (NC), treated with standard diet; hyperlipidemic control (HC), treated with high-fat diet; positive control (PC), treated with high-fat diet and simvastatin (10 mg kg^−1^ d^−1^); L-IPCM-1, M-IPCM-1 and H-IPCM-1, treated with high-fat diet and IPCM-1 at 50, 100 and 200 mg kg^−1^ d^−1^, respectively; L-EPCM-1, M-EPCM-1 and H-EPCM-1, treated with high-fat diet and EPCM-1 at 50, 100 and 200 mg kg^−1^ d^−1^ at 50, 100 and 200 mg kg^−1^ d^−1^, respectively. Values are means ± SD (*n* = 10). #*p* < 0.05 and ##*p* < 0.01, values *versus* NC group values, *p < 0.05 and **p < 0.01, values *versus* HC group values.

Currently, increasing evidences have indicated that edible mushrooms are capable in lowering lipid profile, mainly due to the inhibition of the hepatic HMGR mediated cholesterol synthesis pathway, the elevation of the excretion of bile acids, and the enhancement of the removal of LDL-C from blood into liver mediated by low-density lipoprotein receptor (LDLR).^43–45^ In the present study, we found hypolipidemic effects of IPCM-1 and EPCM-1 were mainly mediated by the regulation of lipid metabolic associated enzymes activities. LPL, known as a serum and liver “cleaning factor”, can catalyze the decomposition of TG in CM and VLDL-C into free fatty acids, and involves in the transformation of dietary lipids into energy for peripheral tissues.^27^ Serum TG is an important source of fatty acids for subsequent oxidation and storage for life maintenance, and this process is closely linked to the functional capability of LPL. Kim *et al.* and Gao *et al.* reported that the hypolipidemic activities of *C. militaris* active constituents were attributed to their beneficial effects on the increase of serum LPL, hepatic lipase and pancreatic lipase activities.^12,20^ In this study, serum LPL activities were measured, and the results are presented in Fig. 8A. Compared with HC mice, when tested at a dose of 200 mg kg^−1^ d^−1^, IPCM-1 supplement remarkably increased the serum LPL activity by 55.93% (*p* < 0.01). Serum LPL activity was dose-dependently and significantly (*p* < 0.01) increased in EPCM-1- treated groups and a maximum of elevation effect by 82.89% was noticed in H-EPCM group related to HC group. Meanwhile, positive simvastatin also effectively increase the serum LPL activity. As expected, we found EPCM-1 and IPCM-1 treatment significantly up-regulated the expression of serum LPL when compared with hyperlipidemic control, and this subsequently decreased serum TG levels effectively *via* the activation of TG hydrolysis pathway.^20,33,46,47^

HMGR is a glycoprotein of the endoplasmic reticulum that participates in the mevalonate pathway, and it is a rate-limiting enzyme in cholesterol synthesis pathway.^48^ Thus, targeting HMGR may serve as a promising therapeutic treatment for hyperlipidemia. In the present study, according to the results of ELISA experiments, we found the hepatic HMGR activity was remarkably lower in PC group (*p* < 0.05), compared with HC mice (Fig. 8B). Interesting, EPCM-1 treatment (50 and 100 mg kg^−1^ d^−1^) could significantly down-regulate the expression of hepatic HMGR as effective as simvastatin, a specific inhibitors of HMGR, related to untreated mice fed with high-fat diet, which was accompanied with the optimal effect in lowering serum TC and LDL-C levels. However, dietary supplement of IPCM-1 did not influence the activity of hepatic HMGR. In this case, we thought that polysaccharides from fermented liquid, rather than mycelia of *C. militaris*, could effectively decrease plasma TC mainly through inhibiting cholesterol synthesis mediated by HMGR,^49^ and LDL-C transport from the serum into hepatocytes may be increased with compensatory up-regulation of LDLR induced by the low cholesterol level in hepatocytes, thereby lowering the serum LDL-C level.^50^

**Fig. 9:**
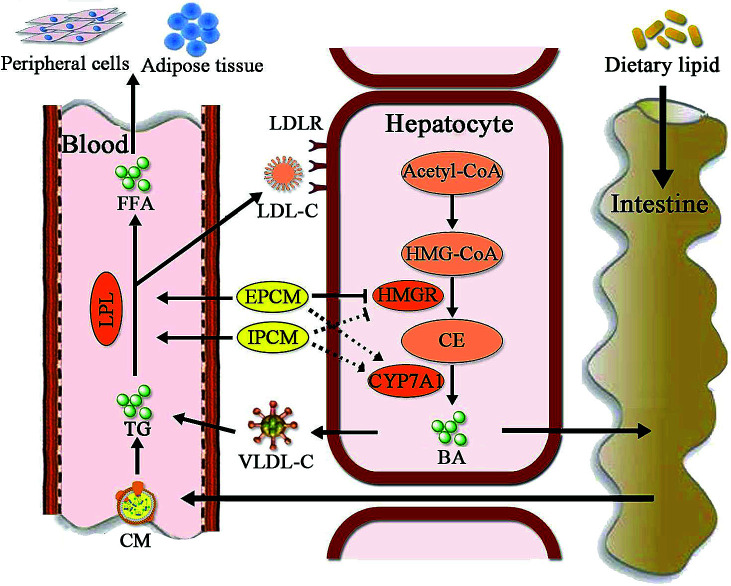
Putative hypolipidemic mechanism of IPCM-1 and EPCM-1 in KM mice fed a high-fat diet. BA, bile acid; CE, cholesterol; CM, chylomicron; CYP7A1, cholesterol 7-alpha-hydroxylase; FFA, free fatty acids; HMG-CoA, 3-hydroxy-3-methyl glutaryl coenzyme A; HMGR, 3-hydroxy-3-methylglutaryl-CoA reductase; LDL-C, low-density lipoprotein-cholesterol; LDLR, low-density lipoprotein receptor; LPL, lipoprotein lipase; TG, triglycerides; VLDL-C, very low density lipoprotein-cholesterol. 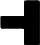 represents the inhibition of polysaccharide on the enzyme; 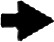 represents the activation of polysaccharide on the enzyme; 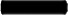 represents the effects of polysaccharide; 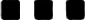 represents no obvious effects of polysaccharide.

Moreover, CYP7A1 is a cytochrome P450 enzyme that catalyzes the rate-limiting reaction in bile acid synthesis pathway and converts cholesterol to 7-alpha-hydroxycholesterol.^51^ Thus, the elevation of hepatic CYP7A1 may be a viable strategy to lowing serum lipids levels. As shown in Fig. 8C, hepatic CYP7A1 activity in simvastatin treated group was slightly increased as compared with HC mice. At the dose we utilized, oral administration of IPCM-1 to high-fat-induced hyperlipidemic mice had no obvious effect in hepatic CYP7A1 activity. EPCM-1 application at 100 mg kg^−1^ d^−1^ caused 15.03% reduction in hepatic CYP7A1 activity with no obvious effect at 50 and 200 mg kg^−1^ d^−1^ related to high-fat diet treated mice. These results revealed that the protective effects of IPCM-1 and EPCM-1 against hyperlipidemia might be attributed to the improvement of serum LPL mediated TG hydrolysis and inhibition of hepatic HMGR mediated cholesterol synthesis pathway (Fig. 9).

As mentioned above, we had successful verified the hypolipidemic activities of IPCM-1 and EPCM-1 in high-fat diet-induced hyperlipidemic mice, nevertheless, this study also has some limitations that need to be considered. Toxicity and maximum tolerated doses of IPCM-1 and EPCM-1 need follow-up research. Further, more in-depth investigations on the potential molecular mechanisms including the gene or protein expression related to hyperlipidemia in liver and intestine of the effects of polysaccharides from *C. militaris* were warranted in the future. Despite these limitations, this study was able to define the protective effects of IPCM-1 and EPCM-1 at different doses on complications related to hyperlipidemia in mice.

## Conclusions

IPCM-1 and EPCM-1 were successfully obtained from *C. militaris* cultivated by submerged fermentation, and were further purified into IPCM-2 and EPCM-2, respectively. Results indicated that the average molecular weight of IPCM-2 and EPCM-2 was 32.5 and 20 kDa, respectively. IPCM-2 and EPCM-2 were mainly composed of mannose, glucose and galactose, and linked by α-glycosidic linkage. In addition, IPCM-1 and EPCM-1, as a dietary supplement, could effectively normalize serum lipid metabolism and ameliorate symptoms of hyperlipidemia. The most effective dose of both IPCM-1 and EPCM-1 was 100 mg kg^−1^ d^−1^, and EPCM-1 exerted stronger hypolipidemic properties than IPCM-1. The protective effects against hyperlipidemia might be attributed to the improvement of serum LPL mediated TG hydrolysis and inhibition of hepatic HMGR mediated cholesterol synthesis pathway. All the results suggested that IPCM and EPCM could be used as potentially natural and functional foods for the prevention and/or alleviation of hyperlipidemia.

## Conflicts of interest

The authors declare that they have no competing interests.

## Abbreviations

AI: Atherosclerosis index
*A*_LPL_
: Activity of serum LPL
ANOVA: Analysis of variance
BA: Bile acids
BSA: Bovine serum albumin
BW: Body weight
CD: Circular dichroism
CM: Chylomicron
*C. militaris*
: *Cordyceps militaris*
CYP7A1: Cholesterol 7-alpha-hydroxylase
DEAE: Diethylaminoethyl
ELISA: Enzyme-linked immunosorbent assay
EPCM: Extracellular polysaccharides of *Cordyceps militaris*
FT-IR: Fourier transform-infrared
GC-MS: Gas chromatography-mass
HC: Hyperlipidemic control
HDL-C: High density lipoprotein-cholesterol
H-IPCM: High-dosage of IPCM
H-EPCM: High-dosage of EPCM
HMG-CoA: 3-Hydroxy-3-methyl glutaryl coenzyme A
HMGR: 3-Hydroxy-3-methylglutaryl-CoA reductase
HPGPC: High performance liquid gel permeation chromatography
HRP: Horseradish peroxidase
IPCM: Intracellular polysaccharides of *Cordyceps militaris*
LDL-C: Lipoprotein-cholesterol
LDLR: Low-density lipoprotein receptor
L-IPCM: Low-dosage of IPCM
L-EPCM: Low-dosage of EPCM
LPL: Lipoprotein lipase
M-IPCM: Moderate-dosage of IPCM
M-EPCM: Moderate-dosage of EPCM
*M*_n_
: Number-average molecular weight
*M*_w_
: Weight-average molecular weight
NAFLD: Non-alcoholic fatty liver disease
NC: Normal control
OD: Optical density
PBS: Phosphate buffered saline
PC: Positive control
SD: Standard deviation
TC: Total cholesterol
TG: Triglyceride
TMB: 3,3′,5,5′-Tetramethylbenzidine
VLDL-C: Very low density lipoprotein-cholesterol

## Supplementary Material

RA-008-C8RA09068H-s001
