# Supplementary material for: Structural characterization of polysaccharides from Cordyceps militaris and their hypolipidemic effects in high fat diet fed mice
Source: RSC Adv. 2018 Dec 7;8(71):41012–22. doi: 10.1039/c8ra09068h (PMC9091693; doi:10.1039/c8ra09068h)
Supplement: RA-008-C8RA09068H-s001 [file RA-008-C8RA09068H-s001.pdf]

Electronic Supplementary Material (ESI) for RSC Advances.
This journal is © The Royal Society of Chemistry 2018

Supporting Information

Structural characterization of polysaccharides from *Cordyceps militaris* and their hypolipidemic effects in high fat diet fed mice

Zhen-feng Huang†, Ming-long Zhang†, Song Zhang*, Ya-hui Wang and Xue-wen Jiang

School of Life Science, South China Normal University, Guangzhou 510631, Guangdong, China

† These authors contributed equally.

*Correspondence author

Materials and methods

Monosaccharide composition analysis

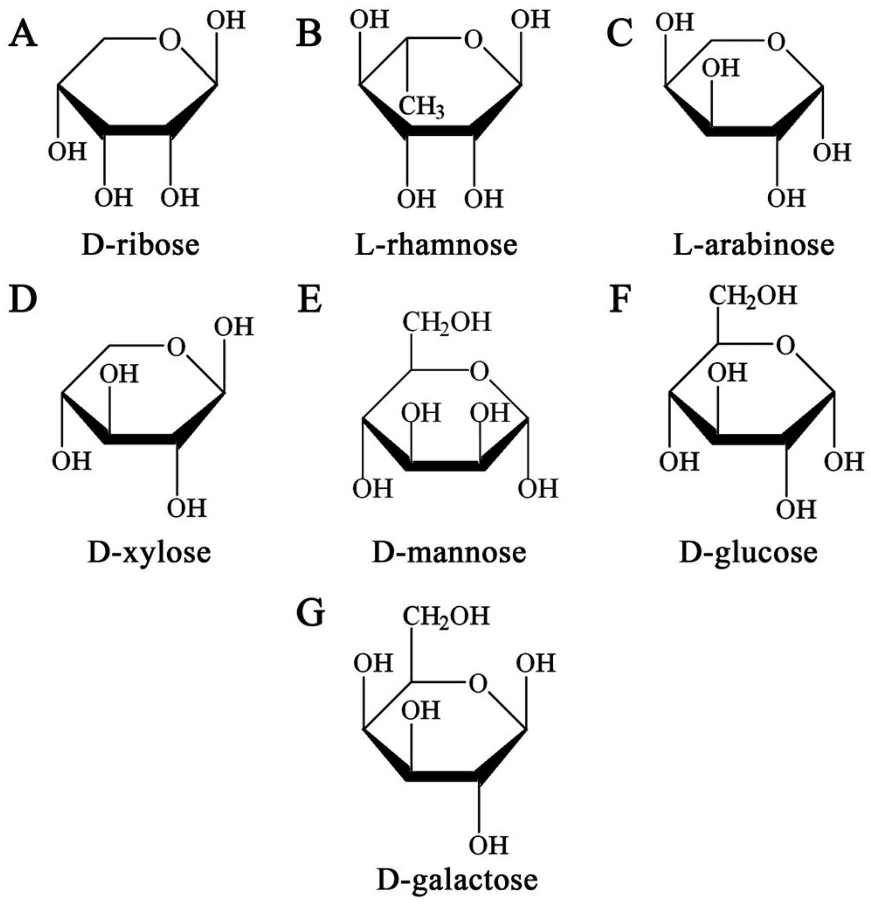

Fig. S1. Chemical structures of monosaccharides used in monosaccaride composition analysis. A. D-ribose, B. L-rhamnose, C. L-arabinose, D. D-xylose, E. D-mannose, F. D-glucose, G. D-galactose.
